# Theoretical study of HgCr_2_Se_3.5_Te_0.5_: a doping-site-dependent semimetal

**DOI:** 10.1038/srep30866

**Published:** 2016-08-02

**Authors:** Xiang-Long Yu, Yuan-Jun Jin, Jiansheng Wu

**Affiliations:** 1 Department of Physics, South University of Science and Technology of China, Shenzhen 518055, P.R. China; 2 School of Physics and Technology, Wuhan University, Wuhan 430072, P.R. China

## Abstract

Weyl semimetals have recently attracted enormous attention due to their unusual features. So far, this novel state has been predicted theoretically and confirmed experimentally in several materials, such as HgTe, LaPtBi, Y_2_Ir_2_O_7_, TaAs, TaP, NbAs, NbP and HgCr_2_Se_4_. Doping plays an important role in the research of condensed-matter materials. However, its influence on the Weyl semimetal has been little investigated. Here, we present detailed first-principles and theoretical studies on HgCr_2_Se_4_ with doping of Te atoms at the Se sites. A special case where only one pair of crossing points locates at the Fermi level is realized in HgCr_2_Se_3.5_Te_0.5_ where one of the Se atoms in the primitive unit cell is replaced by a Te atom. A further study of k·p theory shows that the two points constitute a pair of Weyl nodes with opposite chiralities in the momentum space, and only one edge state and one single Fermi arc are obtained at each boundary of a film. Moreover, through investigations and analyses of different doping cases of HgCr_2_Se_3.5_Te_0.5_, we find that when the type of doping induces inversion symmetry or positional disorder, the Weyl nodes transform into Dirac points resulting in a change from a Weyl semimetal to a Dirac semimetal.

Weyl semimetals are attracting growing interests as a new type of topological quantum state, whose low-energy excitation can be described by a two-component Dirac equation, called the Weyl equation^1^,^2^. In a system with both time-reversal and spacial inversion symmetry, there is at least double degeneracy for each of the energy bands. This leads to a fourfold or multifold degeneracy at one crossing point. Thus there is no Weyl node in such a system. Only when at least one of the two symmetries is broken may Weyl semimetallic states be realized. Weyl semimetals show novel and interesting properties: Weyl nodes and metallic edge states are topologically protected. The latter are the so-called topological edge states and correspond to nonclosed Fermi arcs. The two ends of each Fermi arc connect a pair of Weyl nodes which have opposite chiralities. The detection of Fermi arcs is an effective method to judge whether a semimetal possesses Weyl fermions, and can be performed by angle-resolved photoemission spectroscopy (ARPES) experiments. At present, various Weyl semimetals are predicted theoretically, including HgTe, LaPtBi^3^, Y_2_Ir_2_O_7_^4^, TaP, NbAs, NbP^5^, etc., and the Weyl semimetal TaAs has been confirmed experimentally recently^6^,^7^,^8^,^9^,^10^,^11^. Thereinto, the former two have 4 pairs of Weyl nodes and the others have 12 pairs.

As is well known, doping plays an important role in the research of condensed-matter materials. The effect of doping is usually only to change the carrier concentration. For example, in cuprate materials the doped elements go to the charge resevior layer and the main contribution near the Fermi level is always coming from CuO plane. So doping in the cuprate cases are rather simple and can be understood as a shift of Fermi level^12^,^13^. Besides, doping can also change the electronic structure near *E*_*F*_ dramatically, and the physical properties depend strongly on the doping element, concentration and how the doping takes place. Doping changes the weight of different orbitals near the Fermi surface and might remove or add band crossings, so might change the topology. Hence, the topological properties of the system can be very sensitive to the doping. So far, the influence of doping on the Weyl semimetal has been little investigated, especially the latter doping case. It is desirable to study the associated unusual topological properties in doped systems with the intention of maximizing their potential for novel transport phenomena and possible application in spintronics^14^.

Recently, HgCr_2_Se_4_, as a newly discovered Weyl semimetal, has attracted theoretical 15 and experimental^16^ attentions. It is a ferromagnetic material with the spinel structure^17^, and exhibits novel and unusual properties, including anomalous Hall effect^18^, giant magnetoresistance^19^, red shift of the optical absorption edge^20^, semiconducting character in the paramagnetic state and semimetal character in its ferromagnetic state^19^,^21^,^22^. Its Fermi arc structure has not been confirmed by any ARPES experiments. Theoretical results show that there are two Fermi arcs connecting a pair of Weyl nodes, the Fermi arcs are interrupted by *k*_*z*_ = 0 and a band crossing loop (topologically trivial) in the *k*_*x*_ − *k*_*y*_ plane forms a normal Fermi surface^15^. Since the number of Weyl nodes in HgCr_2_Se_4_ is smaller than that in other predicted Weyl semimetals and it has a relatively simple Fermi surface structure, we take HgCr_2_Se_4_ with Te doping as an example to investigate the influence of doping on Weyl semimetal. We find that the electronic structure near the Fermi level is highly sensitive to Te doping. When the doping concentration is 0.5 (HgCr_2_Se_3.5_Te_0.5_), the compound shows novel and interesting transition for different doping configurations. Simpler Fermi arcs can be realized in the configuration where one of the Se atoms is replaced by a Te atom in the primitive unit cell of HgCr_2_Se_4_. This results in HgCr_2_Se_3.5_Te_0.5_ to only have one edge state and one single Fermi arc at each boundary of a film. This is very different from HgCr_2_Se_4_, which has two edge states and two interrupted Fermi arcs. However, when an inversion-symmetric doping or position-disorder doping case appears, the Weyl nodes will disappear and form Dirac points. Meanwhile, the system will also become into a Dirac semimetal.

## Results and Discussion

In the Brillouin zone of HgCr_2_Se_4_, besides a pair of Weyl nodes, there is a band crossing loop surrounding the Γ point^15^. We first attempt to open the topologically trivial loop. Since the loop is dominantly contributed by Se-4*p* orbitals, we chose congeners of Se (S and Te) to replace the Se atoms so that no free carriers are introduced by this isoelectronic substitution. For the S-doping case, the closed loop can not be opened. However, it can be opened for the Te-doping case due to the more extended wave functions and the larger spin-orbit coupling of Te-5*p* electrons. Especially for the doping concentration of one Te atom per primitive unit cell (HgCr_2_Se_3.5_Te_0.5_), not only one pair of crossing points at *E*_*F*_ can be realized, but also other bands can avoid crossing the Fermi level. Therefore, our subsequent studies focus on the stoichiometric compound HgCr_2_Se_3.5_Te_0.5_.

**Figure 1:**
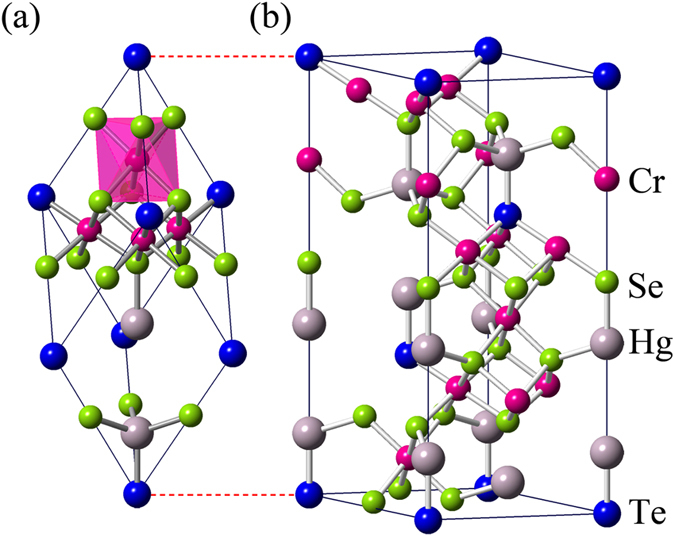
(**a**) Primitive and (**b**) conventional unit cells of HgCr_2_Se_3.5_Te_0.5_ with space group *R*3*M*. Gray, purple, green and blue spheres represent Hg, Cr, Se and Te atoms, respectively. The Cr atom is at the center of Se or Se-Te octahedron.

A primitive unit cell structure of HgCr_2_Se_3.5_Te_0.5_, where one of the Se atoms is replaced by a Te atom in the primitive unit cell of HgCr_2_Se_4_, is shown in Fig. 1(a). This doping configuration is the simplest one of HgCr_2_Se_3.5_Te_0.5_. Both HgCr_2_Se_3.5_Te_0.5_ and HgCr_2_Se_4_ share the same ferromagnetic ground state, so this doping configuration has neither time-reversal symmetry nor spacial inversion symmetry. Fig. 1(b) shows the corresponding conventional unit cell. With respect to HgCr_2_Se_4_, the space group changes to *R*3*m* due to the doping of Te atoms. In the absence of lattice parameters for the true crystal structure, we relaxed the unit cell with consideration of spin polarization. The optimal lattice parameters are *a* = 7.7769 Å, *c* = 19.0496 Å.

**Figure 2:**
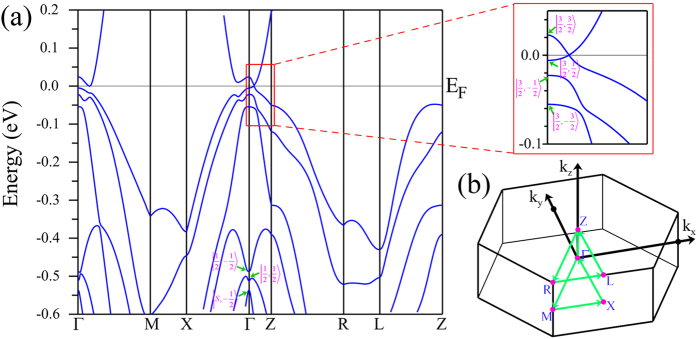
(**a**) The band structures with spin polarization and spin-orbit coupling. Eight low-energy eigenstates at the Γ point are marked. (**b**) Conventional Brillouin zone with *k* path for band structures. The pink dots are the high-symmetry points on the *k* path. Major spin aligns to the coordinate axis of octahedral coordination with its six neighboring anions (Se/Te).

In searching for a perfect Weyl semimetal, we have also studied the compression and stretch effects of HgCr_2_Se_3.5_Te_0.5_. When the structure of the system is optimized as described above, only one pair of Weyl nodes appears in the *k*_*z*_ direction, leading to a Weyl semimetal phase. Through calculating electronic structures with different strains, the results show that the Weyl semimetal phase can be stable in the range of about 0~1% tensile strain. Finally, we find that when the lattice parameters are stretched by 0.7% isotropically, an ideal Weyl semimetal, where there is only one pair of Weyl nodes and no other bands cross *E*_*F*_, can be realized. To our knowledge, there is no straightforward way to apply an isotropic tensile strain, which only has theoretical significance in this study. The two systems without and with strain (0% and 0.7%) have similar band structures and the same topological characters. The difference is that a fraction of the conduction band crosses the Fermi level in the range of less 10 meV for the former case. In order to study the topological properties of the Weyl semimetal conveniently and explicitly, we choose the latter case which presents an ideal Weyl semimetal for detailed calculations. With consideration of spin polarization and spin-orbit coupling, the band structure is calculated and presented in Fig. 2. Since the wave functions of Te-5*p* orbitals are more extended than that of Se-4*p* orbitals, this gives rise to a finite hybridization between Te and its nearest-neighbor Se atoms, the band crossing loop of HgCr_2_Se_4_ around the Γ point is therefore opened at *E*_*F*_. There are no other crossing points except the pair of Weyl nodes at 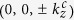 near the Fermi level.

Similar to the case of HgCr_2_Se_4_, each Cr atom lies inside an octahedron composed of Se/Te anions, which leads to a strong octahedral crystal field and a band gap is opened between the *t*_2*g*_ and *e*_*g*_ manifolds. Meanwhile, ferromagnetic state becomes a stable ground state because of the superexchange interactions between the Cr-3*d* electrons. The magnetic moment per Cr atom is about 3*μ*_*B*_ which corresponds to a high spin state. The *p* electrons of the Se and Te atoms are also slightly spin polarized with an opposite moment (about −0.1 *μ*_*B*_ per atom) due to the hybridization with the Cr-3*d* electrons.

We employed similar progresses as that of HgSe, HgTe and HgCr_2_Se_4_^15^,^23^,^24^. The eight energy states at the Γ point near the Fermi level can be identified as 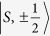, 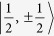, 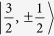 and 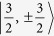 marked in Fig. 2(a). They are linear combinations of atomic orbitals (see the Methods Section for details). In addition, that CdCr_2_S_4_ and CdCr_2_Se_4_ can be well described by local density approximation (LDA) +*U* method has been reported by Fennie and Yaresko *et al.*^25^,^26^. The role that the electronic correlation of the Cr-3*d* electrons plays on the topological properties of HgCr_2_Se_3.5_Te_0.5_ has also been checked. We performed generalized gradient approximation (GGA) +*U* calculations and found that the band inversion and Weyl nodes remain present when the effective Coulomb correlation of Cr-3d electrons is increased to 6 eV.

**Figure 3:**
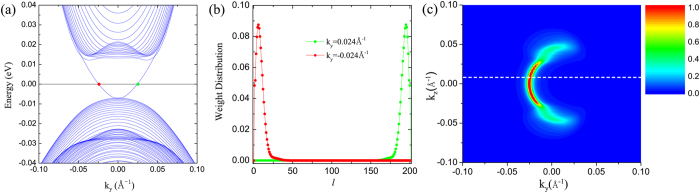
A film with open boundary conditions along *x* direction is considered. The film thickness is 200*a*. (**a**) The band structure with *k*_*z*_ = 0.05*π*/*c*. (**b**) The spacial weight distributions corresponding to two edge states marked by red circle and green square in (a), respectively. *l* is the index of the conventional unit cell along the *x* direction. (**c**) The Fermi arc at the boundary of HgCr_2_Se_3.5_Te_0.5_ for the *k*_*y*_ − *k*_*z*_ side surface. The color represents the normalized spectral weight. The white dashed line corresponds to the left edge state at *E*_*F*_ presented in (**a**).

According to the character of the band structure, an eight-band model of the *k* ⋅ *p* theory is employed for further studies (see the Methods Section for details)^27^. In order to accurately determine the parameters of the model, we have fitted the first-principles energy bands. The fitting parameters are listed in Table 1 and the corresponding band structures are shown in Fig. S2 of Supplementary Materials. Basing on the fitting results, we investigated the edge states in a film. The considered system is finite-sized along *x* direction which is along the conventional lattice vector *a&vec;*. With consideration of open boundary conditions, the edge states and their distributions along the *x* direction can be calculated using the finite difference method^28^,^29^ which is described in the Methods Section. In this finite-length system of HgCr_2_Se_3.5_Te_0.5_, when 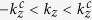, two symmetric bands cross the Fermi level within the bulk gap. From the calculation of the weight distributions of the energy bands at *E*_*F*_,we find that the two bands correspond to two edge states. For example, Fig. 3(a,b) illustrate the edges states along *k*_*y*_ direction with *k*_*z*_ = 0.05*π*/*c* in a film with thickness of 200*a*. Moreover, Weyl semimetals share a basic character that each Fermi arc ends at a pair of Weyl nodes. Therefore, a direct and effective method to detect edge states is via ARPES experiments which can be used to measure Fermi arcs at the boundary. Fig. 3(c) shows the single Fermi arc plotted in the *k*_*y*_ − *k*_*z*_ plane, which ends at 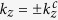. This is very different from normal metals in which the Fermi surfaces must be closed throughout the entire Brillouin zone. It is also different from the result of HgCr_2_Se_4_ where two Fermi arcs end at 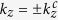 and are interrupted by the *k*_*z*_ = 0 plane. If a single crystalline sample of HgCr_2_Se_3.5_Te_0.5_ can be synthesized, such that a Te atom replaces the same Se site of the primitive cell throughout the lattice, then a simple and clear Fermi arc should be observable by ARPES experiments.

To understand the topological properties qualitatively and capture its essence in HgCr_2_Se_3.5_Te_0.5_, we take a simple and effective 2 × 2 Hamiltonian into account. 
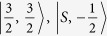
 are chosen as the bases to catch the band-inversion nature, which is similar to that of HgCr_2_Se_4_^15^. The effective Hamiltonian is defined as^30^


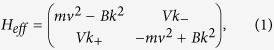


here 
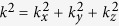
 and *k*_±_ = *k*_*x*_ ± *ik*_*y*_. The energy dispersions for this system are


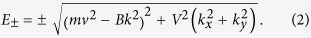


**Figure 4:**
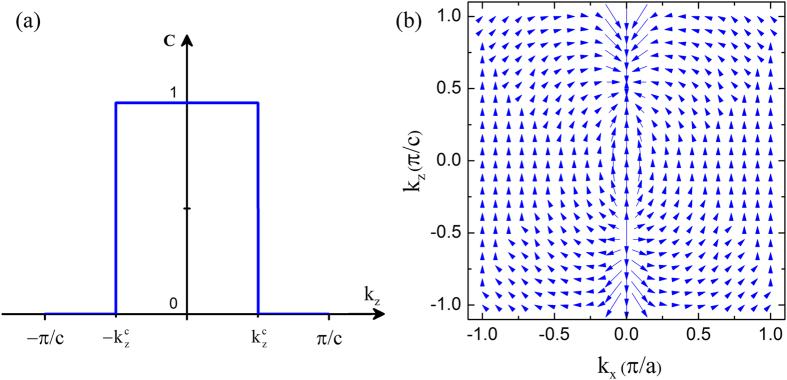
(**a**) Chern Number *C* as a function of *k*_*z*_. (**b**) Berry curvature in the *k*_*x*_ − *k*_*z*_ plane.

With the assumption of *m* > 0 and *B* > 0, two solutions for zero energy gap can be obtained along the Γ − *Z* direction: 
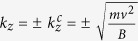
. The two Weyl nodes are located at 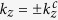, respectively. The Chern number of the entire system is determined by 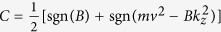, plotted in Fig. 4(a). When 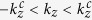, we have a topologically nontrivial phase with *C* = 1; otherwise, *C* = 0, indicating a topologically trivial phase. Therefore, the planes with Weyl nodes are the critical boundaries of the phase transition between *C* = 1 and *C* = 0 planes. These are in good agreement with previous results of the single edge state and the Fermi arc at each boundary (Fig. 3(c)). In the *k*_*x*_ − *k*_*z*_ plane, the Berry curvature displays that the two Weyl nodes have opposite chiralities and form a single pair of magnetic monopoles (Fig. 4(b)), which is the typical feature of a Weyl semimetal.

**Figure 5:**
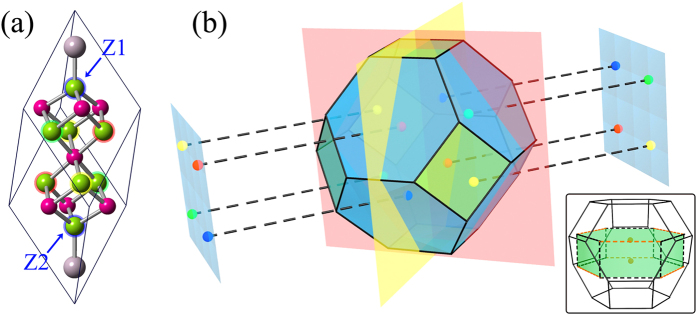
(**a**) With consideration of inversion symmetry, four inequivalent Se atoms for Te doping are marked by red, yellow, green and blue rings in the primitive unit cell of HgCr_2_Se_4_. Z1 and Z2 are a pair of inversion-symmetric Se sites. (**b**) The primitive Brillouin zone of HgCr_2_Se_3.5_Te_0.5_. There are four pairs of Weyl nodes, schematically shown as red, yellow, green and blue circles. They correspond to four Te-doping cases of (**a**), respectively. Inset: The conventional and primitive Brillouin zones are drawn together with dashed and solid lines. The former corresponds to one of four doping cases in (**a**) and a pair of corresponding Weyl nodes are plotted schematically. The connection between the two Brillouin zones are marked by orange dashed lines and the center of the hexagon overlaps with the midpoint of the orange dashed line.

**Figure 6:**
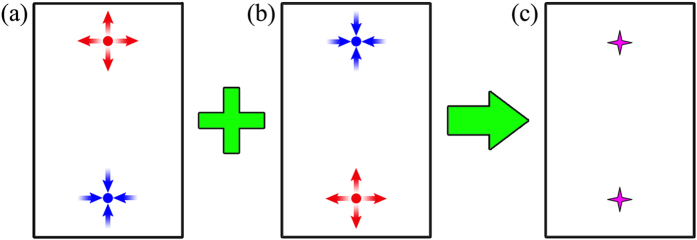
(**a**) Assume that a Te atom is doped at the Z1 position of Fig. 5(a). Schematically, there are two Weyl nodes in the Brillouin zone. Red and blue stars represent a pair of Weyl nodes with opposite chiralities. (**b**) Considering the same lattice vectors as those in (**a**), when a Te atom is doped at the Z2 position instead of the Z1 position, the chiralities of Weyl nodes will exchange. (**c**) When Te atoms are doped at Z1 and Z2 simultaneously in a supercell with spacial inversion symmetry, or Te atoms are doped at the two positions uniformly and randomly with the same doping concentration, the two Weyl nodes at the same *k* point will overlap with each other and form a Dirac point. The arrows denote the chiralities of Weyl nodes in (**a**,**b**). Purple stars represent Dirac points.

There are four inequivalent Se atoms with inversion symmetry in the primitive unit cell(Fig. 5(a)), corresponding to four possible Te-doping scenarios. For each of different cases, the band structures were calculated without considering real-space symmetry (with the space group *P*1), respectively. The calculation results show that each of the doping cases corresponds to a different pair of Weyl nodes. There are four pairs of Weyl nodes in the primitive Brillouin zone, as illustrated in Fig. 5(b). The inset of the figure shows the relation between the conventional (Fig. 2(b)) and primitive Brillouin zones. Meanwhile, we notice that for any one of the four doping cases, an inversion-symmetric doping in a supercell will make the Weyl nodes with opposite chiralities appear at the same *k* point, leading to the formation of Dirac points. The physical mechanism is explained through a schematic diagram in Fig. 6. One can see that when Te atoms are simultaneously doped at inversion-symmetric sites in a supercell (such as X-7 configuration in Fig. S1(b) of Supplementary Materials), two Weyl nodes with opposite chiralities will overlap with each other and form a Dirac point. The electronic structures of three other doping configurations (X-7, X-9 and X-10) have also been investigated in the Supplementary Materials. Based on the analysis in Figs 5 and 6, it can be determined that the systems with X-7 and X-10 configurations are Dirac semimetals with four and two Dirac points,respectively, and the system with X-9 configuration is a Weyl semimetal with four pairs of Weyl node. In addition, it is worth noting that since the atomic properties of Te and Se are very similar and the energy difference between different doping configurations (under consideration in the Supplementary Materials) is small, the Te atoms may be randomly and evenly distributed, leading to a position-disorder type of doping. When this type of doping is present, the Weyl nodes with opposite chiralities will overlap with each other and the crossing points shown in Fig. 5(b) become Dirac points. The topological order and Fermi-arc edge states will also disappear simultaneously, rendering HgCr_2_Se_3.5_Te_0.5_ to change from a Weyl semimetal to a Dirac semimetal.

Through the investigations and analyses of different doping cases of HgCr_2_Se_3.5_Te_0.5_, two significant results can be obtained in the present study: (1) the chiralities and the number of the Weyl points as well as their locations in momentum space depend on the Te-doping sites; (2) in the uniform doping case, both the existence of inversion symmetry and the position-disorder doping can cause that the Weyl nodes with opposite chiralities overlap with each other, leading to the formation of Dirac points.

In summary, we employed first-principles and *k*·*p* methods to investigate the electronic structures and topological properties of HgCr_2_Se_3.5_Te_0.5_ with analyses on each of the cases where Se atoms are substituted with Te atoms in a spatially homogeneous fashion. We find that only one pair of Weyl nodes is present in the entire Brillouin zone, and the results of the Berry curvature and Fermi arc show nontrivial topological properties which are vastly different from that of its parent compound HgCr_2_Se_4_. Equally interesting, when an inversion-symmetric doping or position-disorder doping case is present, the Weyl nodes will disappear and form Dirac points.Meanwhile, the system will also become a Dirac semimetal.

## Methods

### First-principles calculations

In this paper, all of our first-principles calculations for structural optimizations and electronic structures were carried out using the WIEN2K package with a full-potential augmented plane wave^31^, based on the Perdew-Barke-Ernzerhof generalized gradient approximation (PBE-GGA) and its correlation correction (GGA +*U*)^32^. In the GGA +*U* scheme, the effective Coulomb correlation *U*_*eff*_ = *U* − *J* is used, where *U* and *J* are the on-site Coulomb and Hund’s exchange interactions. In addition, the band structure of HgCr_2_Se_3.5_Te_0.5_ have also been investigated with Wu-Cohen-GGA +*U* and hybrid functional^31^,^33^. The two methods show similar results, which are in qualitative agreement with that of PBE-GGA +*U* near the Fermi level. For systems with and without consideration of spatial symmetry during our calculations, the number of *k* points were 3000 and 1000 in the Brillouin zone, respectively.

### Eight-band model from the *k*·*p* theory

With consideration of spin-orbit coupling, the low-energy eigenstates at the Γ point can be constructed through the linear combinations of 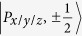, and are given as follows^27^.


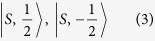



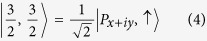



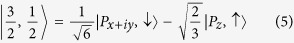



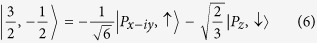



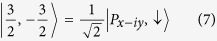



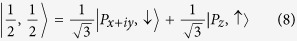



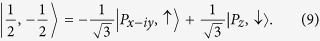


Therefore, the 8 × 8 Kane Hamiltonian can be written as


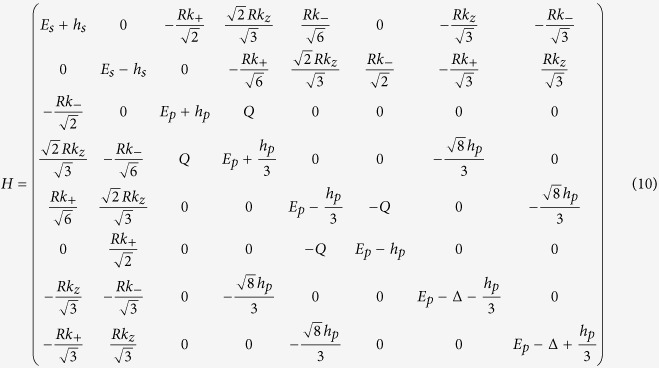


where 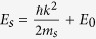, 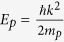 and the physical explanations of the parameters are given in Table 1.

### Finite difference method

The Hamiltonian Eq. (10) with finite size along *x* direction is taken into account. *k*_*y*_ and *k*_*z*_ are good quantum numbers and we replace *k*_*x*_ by 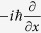. The Hamiltonian can be diagonalized using finite difference method^28^,^29^. We rewrite Eq. (10) in the following way:


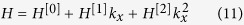


where *H*^[*i*]^ for i = 0, 1 and 2 are give by


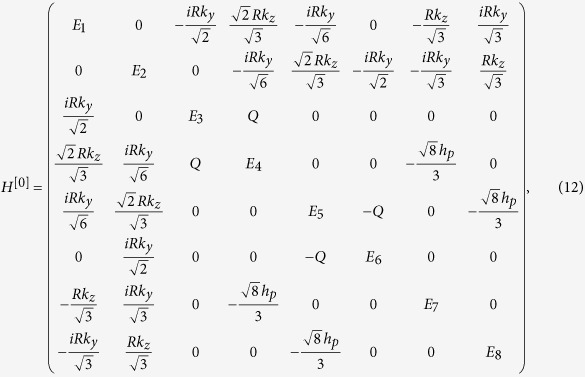



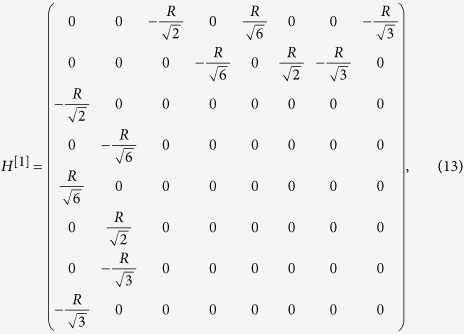



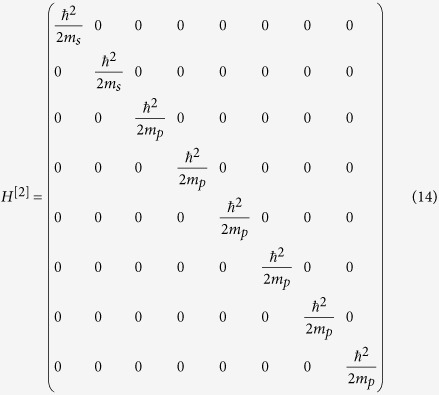


with 
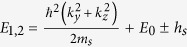
, 
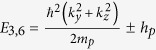
, 
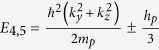
, 
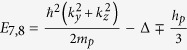
.

Following the discretization scheme of the finite difference method by Xu *et al.*^29^, multi-band Schrödinger equation can be rewritten in a matrix form,


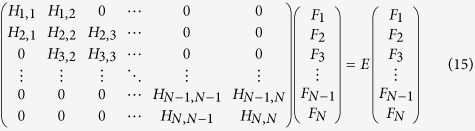


where *N* is the layer number along *x* direction, and *H*_*l*,*l*′_ is a 8 × 8 matrix and corresponds to the interaction between the layer *l* and the layer *l*′. *l* is the layer index. *E* and *F*_*i*_ represent eigenvalue and eigenvector, respectively.


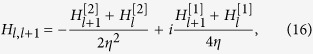



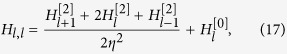



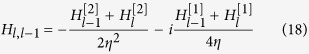


where *η* is the step length along *x* direction.

## Additional Information

**How to cite this article**: Yu, X.-L. *et al.* Theoretical study of HgCr_2_Se_3.5_Te_0.5_: a doping-site-dependent semimetal. *Sci. Rep.* **6**, 30866; doi: 10.1038/srep30866 (2016).

## Supplementary Material

Supplementary Information

## Figures and Tables

**Table 1:** The parameters are fitted to the first-principles calculations through calculating Eq. (10) (see the Methods Section for details).

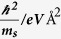	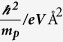	*h*_*s*_/*eV*	*h*_*p*_/*eV*	*E*_0_/*eV*	Δ/*eV*	*R*/*eV*Å	*Q*/*eV*
12.000	−6.4313	0.5281	0.0228	0.0091	0.4822	3.5011	0.0209

*m*_*s*_ and *m*_*p*_ are effective masses of conduction and valence bands, respectively. *h*_*s*_ and *h*_*p*_ describe the exchange splitting, *E*_0_ is the energy difference between conduction and valence bands at the Γ point, Δ is the spin-orbit coupling energy and 
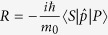
 is the momentum matrix element between conduction and valence bands. Parameter *Q* is introduced to describe the *p*_*x*/*y*_ − *p*_*z*_ hybridization between Te and Se atoms.
