# Supplementary material for: Theoretical study of HgCr2Se3.5Te0.5: a doping-site-dependent semimetal
Source: Sci Rep. 2016 Aug 2;6:30866. doi: 10.1038/srep30866 (PMC4969601; doi:10.1038/srep30866)
Supplement: Supplementary Information [file srep30866-s1.pdf]

Supplementary Materials for "Theoretical study of $HgCr_2Se_{3.5}Te_{0.5}$: a doping-site-dependent semimetal"

Xiang-Long Yu,[1,2] Yuan-Jun Jin,[1] and Jiansheng Wu[1,*]

[1]*Department of Physics, South University of Science and Technology of China, Shenzhen 518055, P.R. China*

[2]*School of Physics and Technology, Wuhan University, Wuhan 430072, P.R. China*

[*]Electronic address: wujs@sustc.edu.cn

In the absence of lattice parameters of $HgCr_2Se_{3.5}Te_{0.5}$ where one of the Se atoms is replaced by a Te atom in the primitive unit cell of $HgCr_2Se_4$, we relaxed the unit cell with consideration of spin polarization. After optimization of the atomic coordinates, two steps are performed for further structural optimization. First, maintaining the value of $c : a$ which is the same to that of $HgCr_2Se_4$[1], we calculated the total energy of $HgCr_2Se_{3.5}Te_{0.5}$ by changing the volume until the minimum-energy volume is obtained (Fig. S 1). The optimized lattice parameters are given in the figure and the corresponding volume of primitive cell is 332.59 Å^3 (2244.43 bohr^3). Second, based on the minimum-energy volume, the value of $c : a$ is optimized, as plotted in the inset of Fig. S1. We find that the difference between the initial value and the optimal value is less than 1%, which is small and can be ignored. Fig. S 2 shows the band structures of $k \cdot p$ and first-preinciples methods along $\Gamma - M/3$ and $\Gamma - Z$. By comparing them near the Fermi level, we can see that they agree well with each other around the Γ point.

The doping configuration that Te atoms are doped into special Se sites has been studied detailedly in the main text. In order to clarify whether the configuration is the lowest-energy one and investigate the influence on Weyl semimetal state with different doping positions, we have considered 25 kinds of doping cases by using supercell with the fixed doping concentration ($HgCr_2Se_{3.5}Te_{0.5}$). All the considered structures are fully optimized by using the Vienna *ab initio* simulation package (VASP)[2]. The exchange-correlation functional within a generalized gradient approximation parametrized by Perdew, Burke, and Ernzerhof (PBE-GGA) has been used[3]. The energy cut-off is set to be 500 eV and the force on each ion is converged to an accuracy 0.02 eV/Å. The schematic configurations and their corresponding total energies are shown in Fig. S 3. Eight Se atoms in the primitive unit cell of $HgCr_2Se_4$ are labeled with Z1(2), A1(2), B1(2) and C1(2). The configurations X-n($n = 1 \sim 11$), Y-1 and Z-1(2) belong to the uniform doping case that each primitive unit cell of $HgCr_2Se_4$ is doped with only one Te atom. The others belong to the nonuniform doping case that some primitive unit cells are doped with more than one Te atom or undoped. The Weyl-semimetal ground state of the configuration X-1 has been confirmed and studied in the main text. However, by comparing the total energies, we can see that the lowest-energy configuration is not X-1 but X-10 among the configurations under consideration. The latter lattice structure is shown in Fig. S 3 (d). Despite this, the configuration X-1 is still of basic importance. Basing on its results, we can further analyze the properties of other

configurations, such as the number and topology of the crossing points near the Fermi level.

We further calculate the electronic structures of X-10 with space group $P2_1/m$. Fig. S 4 shows the band structure and the isoenergic surfaces. There are some crossing points near the Fermi level, but the exact number of the points can not be determined in the band diagram. So the isoenergic surfaces near the Fermi level are checked carefully and the ones with $E = -0.003$ eV and -0.008 eV are plotted in Fig. S 4 (b) and (c), respectively. We can see four small green pockets and yellow pockets in the $k_x - k_y$ plane, and their positions can correspond with each other. Therefore, it can be sure that there are four crossing points near the Fermi level. For this configuration X-10 with A1-B1-B2-A2 doping case, the number of the crossing points is consistent with the analysis of Fig. 5 in the main text. However, these crossing points are not the Weyl nodes. We explain it through a schematic diagram in Fig. 6 of the main text. Since the spacial inversion symmetry is present in the configuration X-10 (Fig. S 3 (d)), the four crossing points are not the Weyl nodes but the Dirac points in the Brilliouin zone.

Although we have considered 25 kinds of configurations with different doping cases and compared their total energies after the full structural relaxation, it is not enough to determine the lowest-energy configuration. Moreover, the atomic properties of Te and Se are very similar and the energy difference between different configurations is small (Fig. S 3 (c)). Therefore, it is highly possible that the compound $HgCr_2Se_{3.5}Te_{0.5}$ is synthesized via uniformly random doping in reality. If this doping case is present, the Weyl nodes will also disappear. Instead, there will be eight Dirac points in Fig. 5 (b) of the main text.

In addition, the electronic structures of the doping configurations X-7 and X-9 (Fig. S 3 (b)) have also been investigated. The energy bands are plotted in Fig. S 5 and Fig. S 6 , respectively. For the configuration X-7 with Z1-Z2 doping case, there are a pair of crossing points along the $\Gamma - Z$ direction in the Brillouin zone. Since the lattice structure has spacial inversion symmetry (space group $C2/m$), based on the analysis in Fig. 6 of the main text, it can be determined that the two crossing points are Dirac points. For the configuration X-9, four Te atoms are respectively doped into Z1, A2, B2 and C2 sites and there are four pairs of crossing points in the Brillouin zone as illustrated in the band structure (Fig. S 6). Because of the lack of inversion symmetry (space group Cm), one can speculate that these crossing points are Weyl nodes. The number of crossing points above has been checked carefully in the entire Brillouin zone. Although these two configurations are not the lowest-energy ones,

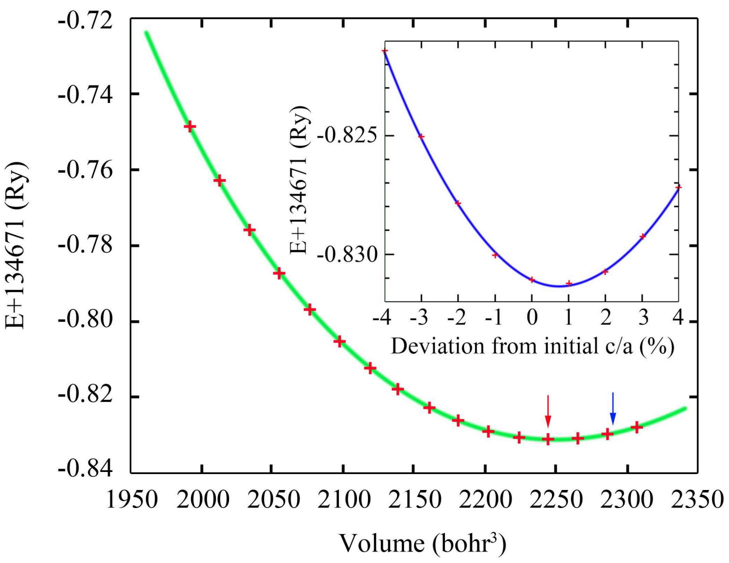

Fig. S 1: (Color online) Structural optimization: Total energies as a function of the volume per primitive unit cell with constant ratio $c : a$ which is the same to that of $HgCr_2Se_4$. The red and blue arrows correspond to the volume of minimum energy (a = 7.7769 Å, c = 19.0496 Å) and the one for following detailed calculations (a = 7.8316 Å, c = 19.1835 Å), respectively. Inset: Total energies as a function of $c : a$ with fixed minimum-energy volume marked by red arrow. 0% corresponds to $c : a$ value of $HgCr_2Se_4$. The optimized value is less than 1%, which is so small that it can be ignored (For the deviation 1%, a decreases by 0.3% and c increases by 0.7%.). The red crosses correspond to our calculated data and the green and blue curves are the fitting results. During the lattice relaxation process, for each lattice structure the energy convergence precision is 0.0001Ry (1.36 meV).

their results of calculations are in agreement with our previous discussion and support our conclusion.

---

[1] Baltzer, P. K., Wojtowicz, P. J., Robbins, M. & Lopatin, E. Exchange interactions in ferromagnetic chromium chalcogenide syinels. *Phys. Rev.* **151**, 367 (1966).

[2] Kresse, G. & Furthmüller, J. Efficient iterative schemes for ab initio total-energy calculations using a plane-wave basis set. *Phys. Rev. B* **54**, 11169 (1996).

[3] Perdew, J. P., Burke, K. & Ernzerhof, M. Generalized gradient approximationmade simple.

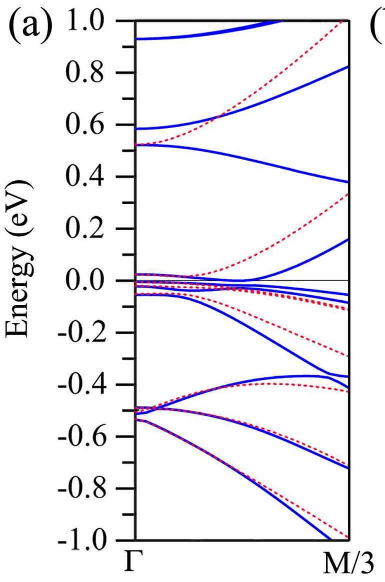

Fig. S 2: (Color online) The band structures of $k \cdot p$ method (red dashed line) compared with the first-principles ones (blue solid line) along $\Gamma - M/3$ (a) and $\Gamma - Z$ (b).

Phys. Rev. Lett **77**, 3865 (1996).

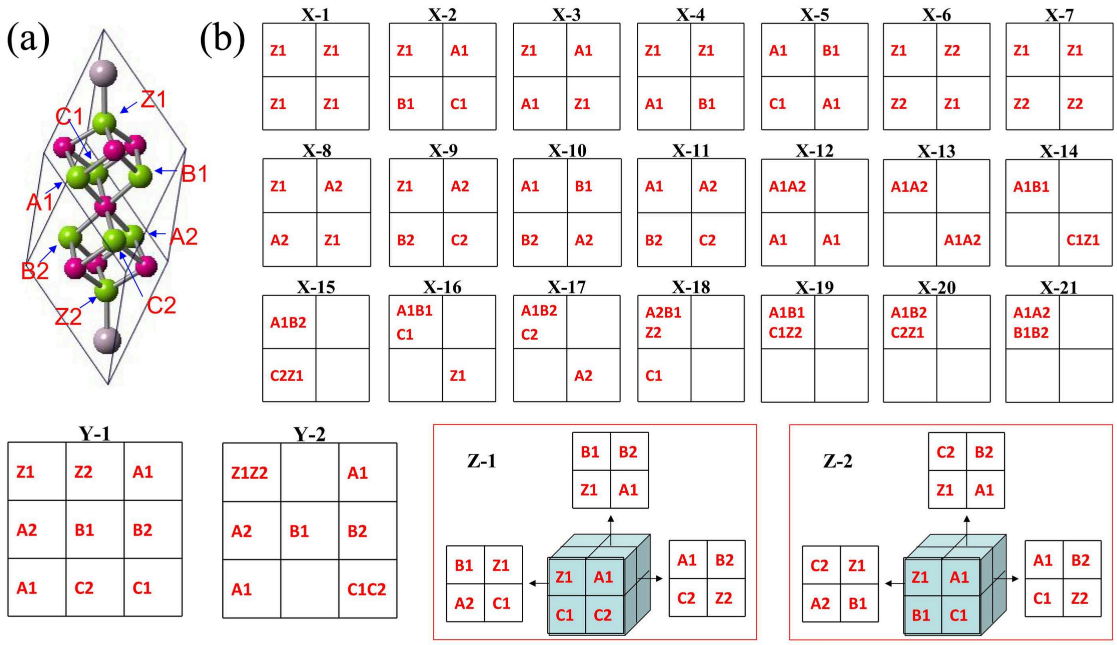

(c)

Configuration	Energy/cell (eV)	Energy/Te (eV)	Configuration	Energy/cell (eV)	Energy/Te (eV)
X-1	-289.66978	-72.41744	X-13	-289.61158	-72.40289
X-2	-289.49862	-72.37466	X-14	-289.68701	-72.42175
X-3	-289.56602	-72.39151	X-15	-289.63509	-72.40877
X-4	-289.64087	-72.41022	X-16	-289.73528	-72.43382
X-5	-289.64205	-72.41051	X-17	-289.70703	-72.42676
X-6	-289.61169	-72.40292	X-18	-289.68826	-72.42207
X-7	-289.69363	-72.42341	X-19	-289.67780	-72.41945
X-8	-289.67853	-72.41963	X-20	-289.76008	-72.44002
X-9	-289.56892	-72.39223	X-21	-289.56583	-72.39146
**X-10**	**-289.77128**	**-72.44282**	Y-1	-650.66741	-72.29638
X-11	-289.51342	-72.37835	Y-2	-651.66541	-72.40727
X-12	-289.65133	-72.41283	Z-1	-579.23825	-72.40478
			Z-2	-579.31516	-72.41439

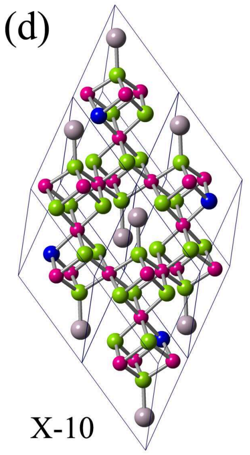

Fig. S 3: (Color online) (a) Gray, purple and green spheres represent Hg, Cr and Se atoms, respectively. Z1(2), A1(2), B1(2) and C1(2) correspond to eight Se sites for Te doping. (b) Three kinds of supercells of $HgCr_2Se_{3.5}Te_{0.5}$ ($2 \times 2 \times 1$, $3 \times 3 \times 1$, $2 \times 2 \times 2$) are constructed and marked by X-n($n = 1 \sim 21$), Y-1(2) and Z-1(2), respectively. Each small black square represents a unit cell of (a), where the text illustrates Te-doping positions. (c) Total energies per supercell and per Te atom of 25 configurations. The lowest-energy configuration is X-10 (bold text). (d) The lattice structure of X-10. The blue sphere represents Te atom.

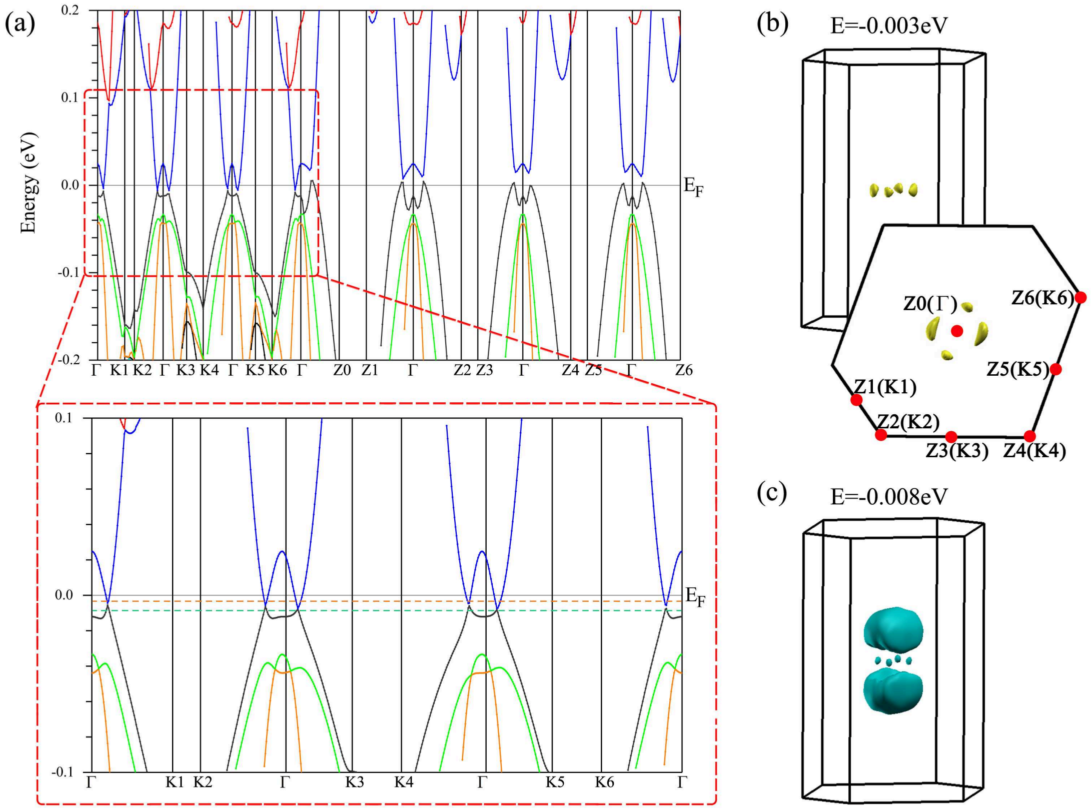

Fig. S 4: (Color online) (a) The band structure of X-10 with consideration of spin polarization and spin-orbit coupling. The lower panel is a partial enlargement of the upper panel with denser k points on the k path. (b) The side and top views of the isoenergic surface in the Brillouin zone with $E = -0.003$ eV which corresponds to the orange dotted line in (a). K1 $\sim$ K6 and Z0 $\sim$ Z6 are high-symmetry points in $k_z = 0$ and $k_z = \pi/c$ planes, respectively. (c) The side view of the isoenergic surface with $E = -0.008$ eV which corresponds to the green dotted line in (a).

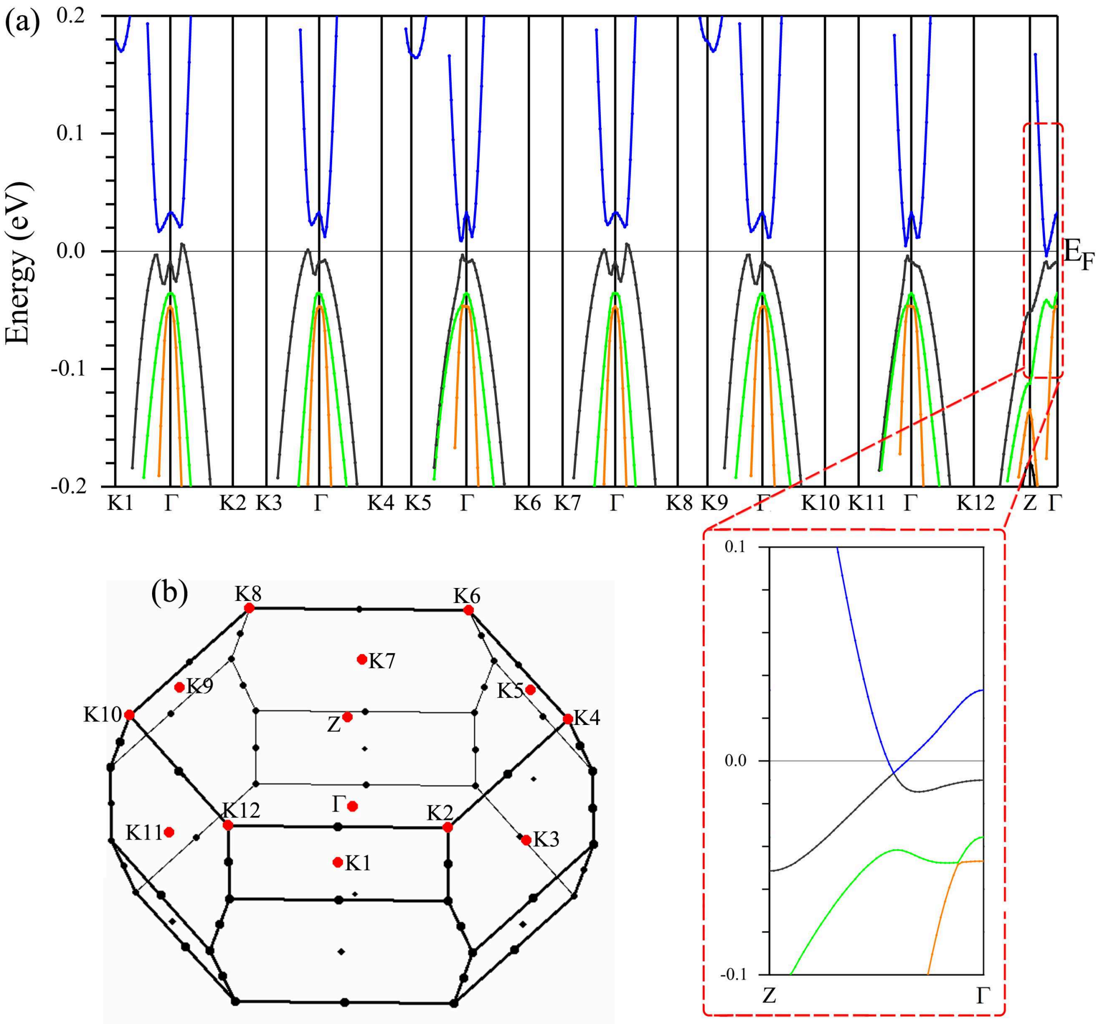

Fig. S 5: (Color online) (a) The band structure of X-7 with consideration of spin polarization and spin-orbit coupling. The lower panel is a partial enlargement of the upper panel with denser k points on the $Z-\Gamma$ path, where there are a pair of crossing points near E_F with consideration of opposite direction. (b) Brillouin zone with high-symmetry points. The red points correspond to the ones on the k path of the band structure.

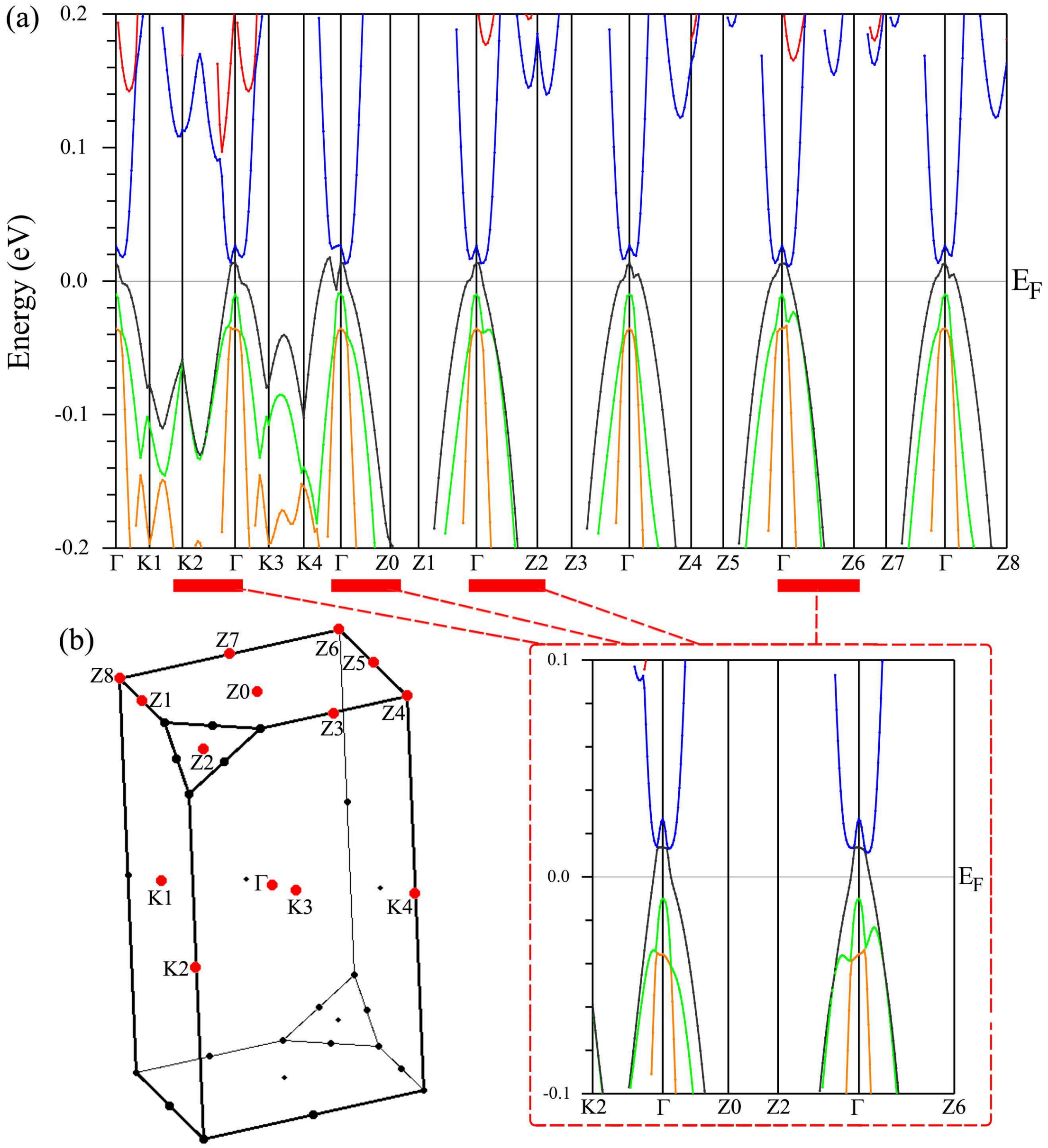

Fig. S 6: (Color online) (a) The band structure of X-9 with consideration of spin polarization and spin-orbit coupling. The lower panel is a partial enlargement of the upper panel with denser k points on the $K2 - \Gamma - Z0 - Z2 - \Gamma - Z6$ path, where there are four pairs of crossing points near E_F with consideration of opposite directions. (b) Brillouin zone with high-symmetry points. The red points correspond to the ones on the k path of the band structure.
